# Genome-wide characterisation of Foxa1 binding sites reveals several mechanisms for regulating neuronal differentiation in midbrain dopamine cells

**DOI:** 10.1242/dev.115808

**Published:** 2015-04-01

**Authors:** Emmanouil Metzakopian, Kamal Bouhali, Matías Alvarez-Saavedra, Jeffrey A. Whitsett, David J. Picketts, Siew-Lan Ang

**Affiliations:** 1 Department of Developmental Neurobiology, NIMR, The Ridgeway, London NW7 1AA, UK; 2 Regenerative Medicine Program, Ottawa Hospital Research Institute, Ottawa, Ontario, CanadaK1H 8L6; 3 Department of Molecular Medicine, University of Ottawa, Ottawa, Ontario, CanadaK1H 8M5; 4 Division of Pulmonary Biology, Cincinnati Children's Hospital Medical Center and University of Cincinnati College of Medicine, Cincinnati, OH 45229, USA; 5 Departments of Biochemistry, Microbiology & Immunology, University of Ottawa, Ontario, CanadaK1H 8M5

**Keywords:** Dopaminergic neuronal differentiation, Foxa1, Foxa2, ChIP-Seq, RNA-Seq, Chromatin, Mouse

## Abstract

Midbrain dopamine neuronal progenitors develop into heterogeneous subgroups of neurons, such as substantia nigra pars compacta, ventral tegmental area and retrorubal field, that regulate motor control, motivated and addictive behaviours. The development of midbrain dopamine neurons has been extensively studied, and these studies indicate that complex cross-regulatory interactions between extrinsic and intrinsic molecules regulate a precise temporal and spatial programme of neurogenesis in midbrain dopamine progenitors. To elucidate direct molecular interactions between multiple regulatory factors during neuronal differentiation in mice, we characterised genome-wide binding sites of the forkhead/winged helix transcription factor Foxa1, which functions redundantly with Foxa2 to regulate the differentiation of mDA neurons. Interestingly, our studies identified a rostral brain floor plate *Neurog2* enhancer that requires direct input from Otx2, Foxa1, Foxa2 and an E-box transcription factor for its transcriptional activity. Furthermore, the chromatin remodelling factor Smarca1 was shown to function downstream of Foxa1 and Foxa2 to regulate differentiation from immature to mature midbrain dopaminergic neurons. Our genome-wide Foxa1-bound cis-regulatory sequences from ChIP-Seq and Foxa1/2 candidate target genes from RNA-Seq analyses of embryonic midbrain dopamine cells also provide an excellent resource for probing mechanistic insights into gene regulatory networks involved in the differentiation of midbrain dopamine neurons.

## Abstract (precis)

**Summary:** ChIP-Seq and RNA-Seq experiments identify novel molecular mechanisms underlying midbrain dopaminergic neuron production downstream of Foxa1 and Foxa2 during mouse neurogenesis.

## INTRODUCTION

The development of midbrain dopaminergic (mDA) neurons from progenitors in the caudal diencephalon and midbrain floor plate is a multistep process involving proliferation, specification, differentiation and axon pathfinding, which ultimately result in the formation of functional dopamine circuits. These brain circuits govern motivated and voluntary motor behaviour and are implicated in neurological disorders such as Parkinson's disease (Lang and Lozano, 1998) and schizophrenia (Sesack and Carr, 2002). The transcription factors and associated signalling molecules that are required for the specification of midbrain dopamine neurons have been extensively studied (Ang, 2006; Prakash and Wurst, 2006; Smidt and Burbach, 2007). Among these transcription factors, Otx2, Foxa1, Foxa2, Lmx1a and Lmx1b are the key players, as they are the earliest to be expressed in midbrain progenitors and they also regulate the expression of signalling molecules, such as Shh (Puelles et al., 2003; Ferri et al., 2007), Fgf8 and Wnt1 (Guo et al., 2007; Yan et al., 2011; Omodei et al., 2008), that are also required for the generation of mDA neurons. Given the numerous cross-regulatory interactions between extrinsic and intrinsic molecules, direct roles can only be revealed through the examination of genomic-wide DNA binding sites of each transcription factor.

Foxa1 and Foxa2 (Foxa1/2) have overlapping roles in the specification and differentiation of mDA neurons (Ferri et al., 2007; Lin et al., 2009). Previous studies have identified gene regulatory networks controlled by Foxa1/2 that are crucial for the specification of mDA neurons (Metzakopian et al., 2012). However, these studies did not reveal how Foxa1/2 govern the differentiation of these neurons at the molecular level because only early proliferating mDA progenitors were assayed in the published ChIP-Seq experiment. Besides Foxa1/2, Otx2 and Lmx1a have also been shown to regulate neuronal differentiation by controlling the expression of the proneural basic helix-loop-helix (bHLH) transcription factor Neurog2 (Vernay et al., 2005; Omodei et al., 2008; Andersson et al., 2006a; Yan et al., 2011; Deng et al., 2011). In turn, Neurog2 and another bHLH transcription factor, Ascl1, function cooperatively to regulate neuronal differentiation of mDA progenitors in the ventral midbrain, between E10.5 and E14.5 (Kele et al., 2006; Andersson et al., 2006b). Loss of Neurog2 leads to loss of about 60% of the mDA neurons, whereas double mutants of *Neurog2* and *Ascl1* exhibit complete loss of all mDA neurons in mice (Andersson et al., 2006a; Kele et al., 2006). Despite the important role of Neurog2 in mDA neurogenesis, how its expression is activated in the midbrain remains unknown.

We therefore set out to discover the molecular mechanisms of how Foxa1/2 regulate neurogenesis in mDA cells by performing ChIP-Seq for these transcription factors on embryonic day (E) 12.5 mDA progenitors and neurons. Our results identified a rostral brain floor plate *Neurog2* enhancer that requires direct input from Otx2, Foxa1/2 and an E-box transcription factor for its transcriptional activity. In addition, we found that Foxa1/2 directly regulate the expression of an epigenetic regulator, Smarca1 (also known as Snf2l). Smarca1, an ISWI chromatin remodelling protein with multiple functions including nucleosome assembly/spacing during replication and transcriptional regulation, is known to regulate neuronal differentiation through repression of expression of the transcription factor Foxg1 (Yip et al., 2012). Recently, Smarca1 has been shown to compensate for another closely related member of the ISWI family, Smarca5 (also known as Snf2h), in regulating Purkinje and granule neuron progenitor expansion in the developing cerebellum of mice (Alvarez-Saavedra et al., 2014). However, the role of Smarca1 in the midbrain has not yet been investigated. In this study, we also demonstrate a novel role for Smarca1 in promoting mDA neurogenesis.

In summary, our genome-wide studies of Foxa1 in embryonic mDA progenitors and neurons successfully identified an important regulatory enhancer and a novel chromatin factor facilitating mDA neurogenesis. These results attest to the high quality of our genome-wide data set that will serve as an excellent resource for mechanistic insights into the process of mDA neuronal differentiation.

## RESULTS

### Genome-wide binding sites for Foxa1 and Foxa2 in E12.5 mDA cells

**Fig. 1.**
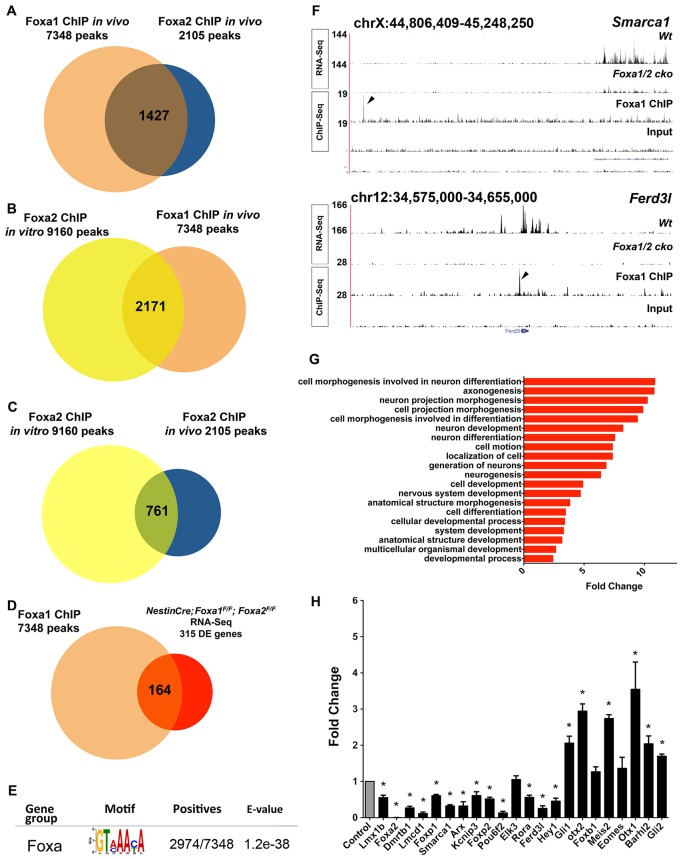
**Foxa1 directly regulates genes with functions in neuronal differentiation.** (A) Overlap between *in vivo* Foxa1 and *in vivo* Foxa2 ChIP-Seq data. (B) Overlap between *in vitro* Foxa2 and *in vivo* Foxa1 ChIP-Seq data. (C) Overlap between *in vivo* and *in vitro* Foxa2 ChIP-Seq data. (D) The overlap between genes bound by Foxa1 and differentially expressed in mDA cells of *Foxa1/2* cko embryos at E12.5. (E) Foxa motifs are enriched in Foxa1-bound genomic regions. (F) Genomic regions around two Foxa1 targets: *Smarca1* and *Ferd3l*. Arrowheads indicate the Foxa1-bound region validated in H. (G) Enrichment of Gene Ontology (GO) terms for biological processes associated with downregulated genes bound by Foxa1. (H) qRT-PCR analyses of transcriptional regulators identified by ChIP-Seq and RNA-Seq using ventral midbrain tissue from E12.5 control and *Foxa1/2* cko embryos. Expression of all genes in mutant and control tissues is normalised relative to the levels in control tissues (set to 1; control bar). *P<0.05 (two-tailed *t*-test). Error bars indicate s.e.m.

We performed genome-wide characterisation of Foxa1 and Foxa2 target genes by ChIP-Seq of mouse ventral midbrain at E12.5, which includes both mDA neurons and progenitors, in order to identify target genes that regulate mDA neuronal differentiation. ChIP-Seq experiments of E12.5 mDA cells were performed with both Foxa1-specific and Foxa2-specific antibodies. Since peak calling using Galaxy (usegalaxy.org) identified 7348 Foxa1-bound regions (supplementary material Table S1) with FDR <5%, whereas the Foxa2-specific antibody identified only 2105 regions (supplementary material Table S2), we decided to use the Foxa1 ChIP-Seq data set for downstream analysis. 1427 of the 2105 (68%) Foxa2-bound regions overlapped with Foxa1-bound regions from the *in vivo* ChIP-Seq experiments with Foxa2 and Foxa1 antibodies, respectively, confirming high-quality reproducible datasets (Fig. 1A; supplementary material Table S2). 2171 Foxa1-bound regions overlapped with Foxa2-bound regions in mDA progenitors from *in vitro* generated mDA progenitors by differentiation of mouse embryonic stem cells (ESCs), as published previously (Fig. 1B; supplementary material Table S3) (Sesack and Carr, 2002). In addition, we discovered that 761 *in vivo* Foxa2-bound regions overlapped with the *in vitro* Foxa2-bound regions (Fig. 1C). Since the Foxa1 ChIP-Seq experiment provided many more putative downstream bound regions for validation, we focused on this data set for downstream analysis. We also overlapped the list of genes associated with these common Foxa1-bound and Foxa2-bound regions with a list of 444 genes that are specifically expressed in the midbrain floor plate, as generated by expression profiling of E10.5 embryos (Gennet et al., 2011). Interestingly, 91 of these floor plate genes were bound by Foxa1, suggesting that Foxa1, like Foxa2, directly regulates the expression of many genes in this tissue (supplementary material Fig. S1, Table S3) (Sesack and Carr, 2002).

To identify functionally relevant target genes, we next overlapped the ChIP-Seq data set with a list of genes differentially expressed in E12.5 mDA cells in the presence or absence of Foxa1/2. The latter gene list was derived by using RNA-Seq to compare the transcriptome of mDA cells in the ventral midbrain of control versus *NestinCre;Foxa1^flox/flox^;Foxa2^flox/flox^* double-mutant embryos (referred to as *NestinCre;Foxa1/2* cko) at E12.5. In contrast to previously published work in which the *Foxa1^l^^acZ/lacZ^;NestinCre;Foxa2^flox/flox^* mutants analysed (Ferri et al., 2007) had a null deletion of *Foxa1* and a conditional deletion of *Foxa2*, both genes are conditionally deleted in all mDA progenitors by Cre recombinase in *NestinCre;Foxa1/2* cko embryos at E11.5. Both mutants show a similar reduction in the number of Nurr1^+^ postmitotic mDA neurons and a block in differentiation of Nurr1^+^ immature to Nurr1^+^ Th^+^ mDA neurons (supplementary material Fig. S2).

Combining the associated Foxa1-bound genes and RNA-Seq data from wild-type versus *NestinCre;Foxa1/2* cko embryos revealed 164 differentially expressed and Foxa1-bound genes (Fig. 1D; supplementary material Table S5). ChIP-Seq and RNA-Seq data of *Smarca1* and *Ferd3l* are shown in Fig. 1F to illustrate the quality of the data. De novo motif analyses of the Foxa1-bound regions using MEME (Bailey and Elkan, 1994) revealed a substantial enrichment in Foxa motif sites (Fig. 1E).

We then surveyed the functional annotations of these putative Foxa1/2 targets using Gene Ontology (GO). A wide spectrum of biological processes were significantly enriched, including neuron differentiation, neuron projection morphogenesis and axon guidance, that are consistent with established roles for Foxa1/2 in mDA neurons (Fig. 1G; supplementary material Table S5).

We also carried out independent validation of the group of differentially expressed genes classified as transcriptional regulators using RT-qPCR assays on independent samples of ventral midbrain tissue dissected from *NestinCre;Foxa1/2* cko and control embryos at E12.5. 19/22 genes were confirmed to be differentially expressed (Fig. 1H). Altogether, these results provide initial validation of the high quality of the combined list of Foxa1-bound regions and Foxa1/2-regulated genes.

### A Foxa1-bound *Neurog2* enhancer is co-regulated by homeodomain and E-box-binding transcription factors

**Fig. 2.**
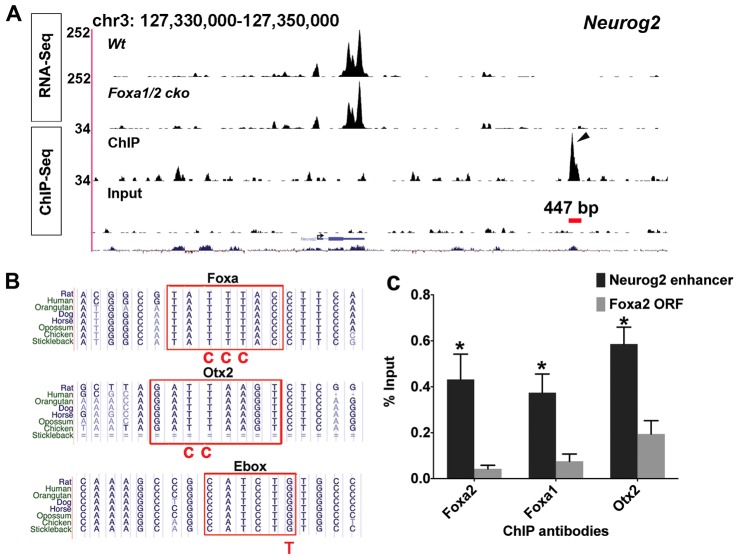
**Identification of a novel enhancer for *Neurog2* that is active in mDA cells at E12.5.** (A) Foxa1 ChIP-Seq identifies a peak over a conserved region downstream of *Neurog2* (arrowhead). (B) Multiple species alignment of the *Neurog2* enhancer provided by the UCSC genome browser. The sequences of the Foxa, Otx2 and E-box DNA binding motifs are highly conserved; the nucleotide substitutions in the mutated version of the motifs are indicated in red. (C) ChIP-qPCR data indicate that Foxa1/2 and Otx2 directly bind to the *Neurog2* enhancer region (red bar in A). *P<0.05 (Student's *t*-test). Error bars indicate s.e.m.

**Fig. 3.**
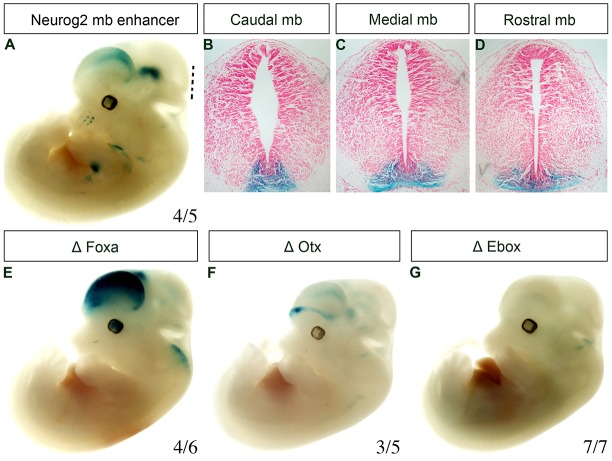
**The Foxa, Otx2 and E**-**box motifs are essential for proper function of the *Neurog2* midbrain enhancer.** (A) E12.5 transgenic mouse embryo expressing *lacZ* (blue) from a minimal promoter and the candidate *Neurog2* midbrain (mb) enhancer bound by Foxa1. The dashed line indicates plane of sectioning. (B-D) Histological sections through the midbrain of the transgenic embryo shown in A. These sections have been counterstained with Nuclear Fast Red. (E-G) E12.5 transgenic mouse embryos expressing *lacZ* from a minimal promoter and the candidate *Neurog2* enhancer with mutations in the (E) Foxa, (F) Otx2 and (G) E-box motifs. (A,E-G) The number of embryos exhibiting *Neurog2* enhancer reporter activity over the total number of transgenic embryos is indicated for each construct.

Neurogenesis in the midbrain requires activation of Neurog2 in floor plate progenitors, as previously shown using loss-of-function studies of Neurog2 in mice (Andersson et al., 2006a; Kele et al., 2006). Upstream of *Neurog2*, the genes *Foxa1/2*, *Otx2* and *Lmx1a* are also required for the neurogenesis of mDA neural progenitors (Ferri et al., 2007; Yan et al., 2011; Omodei et al., 2008; Andersson et al., 2006a,b; Vernay et al., 2005). *Lmx1a* has previously been suggested to regulate neurogenesis in an indirect manner through regulation of the downstream gene *Msx1* (Lin et al., 2009; Andersson et al., 2006b). By contrast, the identification of a Foxa1-bound peak downstream of *Neurog2* coding sequences (*Neurog2* 3′) raised the possibility that Foxa2 might directly activate *Neurog2* expression through this putative enhancer region in embryonic mDA progenitors (Fig. 2A). A conserved 447 bp genomic fragment within this *Neurog2* 3′ Foxa1-bound peak showed enhancer activity specifically in midbrain floor plate and also in the cerebral cortex, when analysed in mouse transient transgenic experiments at E12.5 (Fig. 3A-D). This enhancer is distinct from previously identified enhancers of *Neurog2* (Scardigli et al., 2001). Analysis of the sequences within this genomic fragment using the software package Tess (http://www.cbil.upenn.edu/tess) revealed, as expected, one putative forkhead binding site; however, the presence of highly conserved binding sites for homeodomain and bHLH transcription factors within this enhancer (Fig. 2B) also suggested direct roles for such transcription factors in regulating the expression of *Neurog2*.

We first confirmed by independent ChIP-qPCR using chromatin from different E12.5 ventral midbrain embryonic mouse tissue that Foxa1, Foxa2 and Otx2 bound to the *Neurog2* enhancer (Fig. 2C). Next, we mutagenised the binding motifs of these transcription factors separately in two different constructs, and showed that these mutations led to loss of expression of β-galactosidase expression in a transient transgenesis assay using Foxa and Otx2 binding site mutant *Neurog2* enhancer-*lacZ* reporter constructs (Fig. 3E,F). Similarly, mutation of the E-box binding motif abrogated *lacZ* expression in the floor plate (Fig. 3G). Altogether, these data identified a novel *Neurog2* enhancer, the activity of which in mDA progenitors requires binding of Otx2, Foxa and E-box-binding transcription factors.

### Identification of novel Foxa1-regulated transcription factors expressed in mDA cells

We have previously suggested that Foxa1/2 transcription factors might act in a feed-forward manner to control gene regulatory networks in mDA progenitors (Lin et al., 2009; Andersson et al., 2006b). To investigate this hypothesis, we focused on Foxa1-bound and Foxa1/2-regulated targets that are classified as transcriptional regulators to identify novel genes that may act as co-factors of Foxa1/2 in transcriptional regulation. We focussed on the validated downregulated genes first (Fig. 1D), since previous studies showed that more downregulated than upregulated genes positively regulate mDA progenitor identity (Metzakopian et al., 2012). Among the 13 Foxa1-bound and Foxa1/2 downregulated genes in *Nestin;Foxa1/2* cko embryos (supplementary material Table S5), five previously known Foxa1/2 downstream target genes, namely *Ferd3l* (also known as *Nato3*), *Foxa1*, *Foxa2*, *Lmx1b* and *Nurr1* (also known as *Nr4a2*), already have defined roles in mDA neurogenesis (Yan et al., 2011; Ono et al., 2010; Deng et al., 2011; Zetterström et al., 1997). *Foxp1* has also previously been shown to promote mDA neuron development during the differentiation of mouse ESCs (Konstantoulas et al., 2010).

**Fig. 4.**
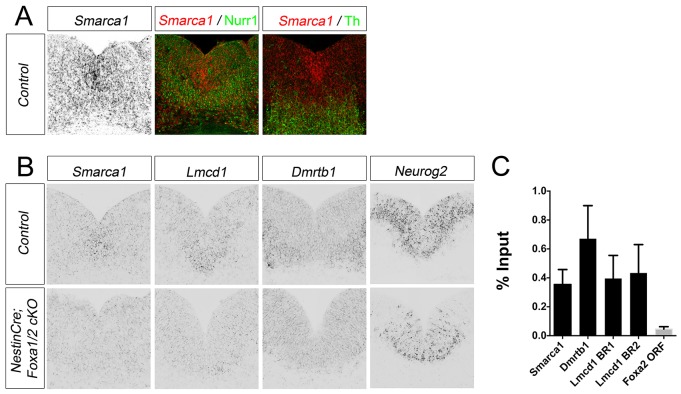
**Expression of novel transcriptional regulators *Smarca1*, *Dmrtb1*, *Lmcd1* and *Neurog2*, that are bound by Foxa1 and regulated by Foxa1/2 in the ventral midbrain.** (A,B) Section *in situ* hybridisation on coronal sections of wild-type and *NestinCre;Foxa1/2* cko mouse embryos at E12.5. *Smarca1* expression is pseudo-coloured red in the middle and righthand images in A, where it is shown together with immunostaining for Nurr1 and Th in postmitotic mDA neurons. (C) Independent ChIP-qPCR experiments were performed with chromatin prepared from E12.5 ventral midbrain tissue for *Smarca1*, *Dmrtb1* and two bound genomic regions associated with *Lmcd1* termed BR1 and BR2. Error bars indicate s.e.m.

We performed *in situ* hybridisation experiments to determine the expression patterns of three novel differentially expressed genes: *Smarca1*, *Dmrtb1* and *Lmcd1*. *Smarca1* was expressed widely in all cells, but with stronger expression in mDA progenitors in the floor plate of mouse embryos at E12.5 (Fig. 4A). This result is consistent with previous studies showing that *Smarca1* is widely expressed throughout the mouse embryo at low levels by radioactive *in situ* hybridisation on sagittal sections from E9.5 to E15.5 (Lazzaro and Picketts, 2001). Expression of *Smarca1* co-localised with Nurr1 and Th as detected by immunostaining in postmitotic mDA neurons (Fig. 4). *Smarca1* expression was specifically downregulated in mDA progenitors in mutant embryos, whereas expression in other surrounding midbrain cells appears unaffected (Fig. 4B). *Lmcd1* was more strongly expressed in basal and floor plate midbrain progenitors than in other midbrain cells and its expression was also downregulated in mDA progenitors in mutant embryos at E12.5 (Fig. 4B). By contrast, *Dmrtb1* showed higher expression in postmitotic mDA neurons than in mDA progenitor cells and expression was diminished in postmitotic neurons in the ventral midbrain of E12.5 embryos (Fig. 4B).

Since there is a neuronal differentiation phenotype in *NestinCre;Foxa1/2* cko mutants (supplementary material Fig. S2), we also analysed *Neurog2* expression by *in situ* hybridisation. *Neurog2* expression was reduced in mDA progenitors in the floor plate and almost completely abolished in the basal progenitors of mutant embryos (Fig. 4B), consistent with published findings (Andersson et al., 2006b). Surprisingly, *Neurog2* differential expression was below the statistical threshold to be detected in our RNA-Seq data.

In summary, analyses of Foxa1 targets resulted in the identification of several novel transcription factors that are expressed in mDA progenitors and/or neurons at E12.5 with expression patterns that are modified in the absence of Foxa1/2. The Foxa1-bound regions associated with these genes were independently validated by ChIP-qPCR analyses (Fig. 4C).

### Loss of function of Smarca1 leads to delayed maturation of mDA neurons

**Fig. 5.**
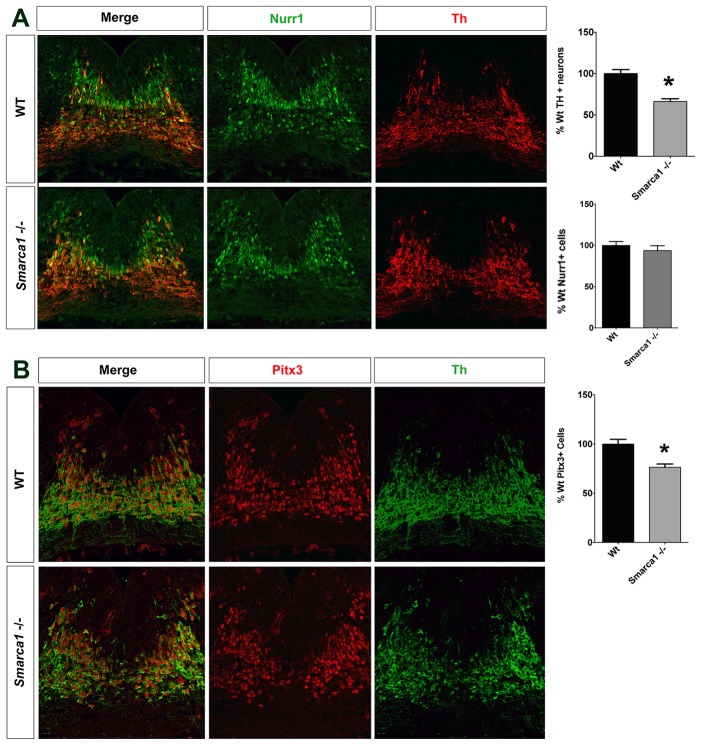
**Smarca1 is required for maturation of a subset of mDA neurons.** (A,B) Whereas the total number of Nurr1^+^ mDA neurons remains unchanged, the numbers of mature Th^+^ and Pitx3^+^ mDA neurons are reduced by ∼25% in the *Smarca1* mutant at E12.5. The average±s.e.m. of at least three independent replicates is shown. *P<0.05 (two-tailed *t*-test).

Given recent studies suggesting a role of epigenetic factors in the regulation of mDA neuronal differentiation (reviewed by van Heesbeen et al., 2013; Yi et al., 2014), we focused on the role of Smarca1 in the differentiation of mDA neurons. Smarca1 has previously been shown to promote the differentiation of neuronal progenitors in the cortex (Yip et al., 2012), but its role in the midbrain was not examined. We generated *Smarca1^−/−^* embryos to determine whether it has a role in the development of mDA neurons (Fig. 5).

**Fig. 6.**
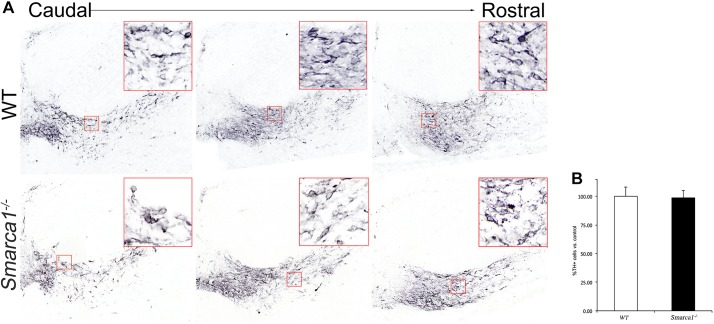
**The neuronal differentiation defect in *Smarca1* mutant embryos is recovered by E16.5.** (A) Three representative coronal midbrain sections matched for position along the anterior-posterior axis between mutants and wild-type embryos, which were used to quantify the number of Th^+^ mDA neurons. (B) The average number of Th^+^ neurons in mutants (*n*=4) was divided by the average number of Th^+^ neurons in wild-type embryos (*n*=5) and represented as percentage±s.d.

We first determined the status of immature and mature mDA neurons using double immunohistochemical labelling with the orphan nuclear hormone receptor Nurr1 and the rate-limiting enzyme for dopamine synthesis tyrosine hydroxylase (Th). Nurr1 labels both immature and mature mDA neurons, whereas Th specifically labels mature mDA neurons. Although the total numbers of Nurr1^+^ mDA neurons remained unchanged, a 25% reduction in the number of mature Nurr1^+^ Th^+^ mDA neurons was observed in *Smarca1* mutant compared with wild-type embryos at E12.5 (Fig. 5A). We confirmed the reduction in mature mDA neuronal number by determining the expression of Pitx3, another marker of mature mDA neurons. Accordingly, a similar reduction in the number of Pitx3^+^ mature mDA neurons was observed in mutant versus wild-type embryos (Fig. 5B). By contrast, we could not detect any significant change in Th^+^ mature mDA neuronal cell numbers by immunohistochemistry on coronal sections of mouse embryos at E16.5 (Fig. 6).

These results indicate that there is a partial delay in the maturation of mDA neurons in *Smarca1* mutants at E12.5, which appears to be rescued by E16.5. The defect probably occurs during the differentiation from immature to mature mDA neurons, as no obvious changes in the expression of progenitor markers, such as Sox2, Lmx1a, Lmx1b, *Neurog2* and *Corin*, could be detected in *Smarca1* mutants as compared with control embryos (supplementary material Fig. S3).

### Smarca1 promotes the differentiation of mDA neurons from mouse epiblast stem cells

**Fig. 7.**
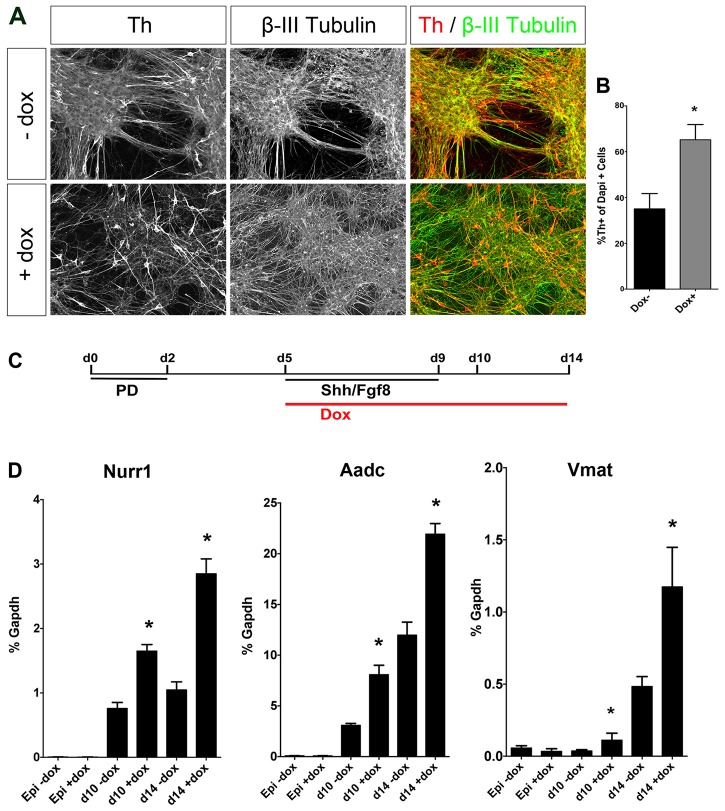
**Overexpression of Smarca1 induces a higher efficiency of mDA neuron generation *in vitro*.** (A) Immunostaining of day (d) 14 cells after monolayer differentiation as derived from rtTA Smarca1 epiblast stem cells (EpiSCs). (B) Quantification of the immunostaining in A, illustrating the percentage of DAPI-stained cells expressing Th in doxycycline (Dox)-treated and untreated cultures at d14 following monolayer differentiation. Mean±s.e.m. of three independent replicates. (C) The *in vitro* differentiation procedure. EpiSC monolayer cultures were exposed to the fibroblast growth factor inhibitor PD0325901 (PD) from d0 to d2. (D) qRT-PCR analyses of *Nurr1*, *Aadc* and *Vmat2* in EpiSCs, d10 and d14 cultures after monolayer differentiation of rtTA Smarca1 EpiSCs with or without Dox treatment. Error bars indicate s.e.m. (B,D) *P<0.05 (two-tailed *t*-test).

Given the possibility of redundant functions of different members of the SWI/SNF family that would mask the roles of any single member, we performed gain-of-function studies of Smarca1 function using a mouse epiblast stem cell differentiation protocol to generate mDA neurons (Jaeger et al., 2011). We first generated stably transfected ESCs carrying a doxycycline-inducible *Smarca1* expression construct. These *Smarca1* gain-of-function ESCs were differentiated first into epiblast cells. These cells were then grown in differentiation medium alone or in the presence of doxycycline so that *Smarca1* expression was induced in postmitotic mDA neurons at day 5 of monolayer differentiation, and the cells were then cultured to day 14 to generate βIII-tubulin^+^ Th^+^ neurons (Fig. 7A-C). As expected, some Th^+^ neurons (37%) were observed in the parental or non-induced control cultures (Fig. 7A,B). By contrast, a higher proportion of Th^+^ neurons (62%) differentiated in monolayer cultures overexpressing Smarca1 (Fig. 7A,B). Most of the Th^+^ neurons were found in large clusters and had a mature neuronal phenotype (Fig. 7A). We also found by RT-qPCR that the expression of other mature mDA neuron markers, such as *Nurr1*, *Aadc* (*Ddc*) and *Vmat2* (*Slc18a2*), was also enhanced in cultures with induced Smarca1 expression compared with control cultures (Fig. 7D).

Altogether, these results show that Smarca1 increases the number of mature mDA neurons generated by the differentiation of epiblast stem cell cultures, indicating a role in promoting mDA neuronal differentiation *in vitro*.

## DISCUSSION

### Identification of Foxa1-bound and regulated genes in E12.5 mDA cells

We have previously published a list of candidate Foxa1/2-bound and regulated genes in mDA progenitors generated by *in vitro* differentiation of mouse ESCs (Metzakopian et al., 2012). Here, we identified candidate target genes bound and regulated in mouse embryonic mDA progenitors and neurons at E12.5, which we will refer to as the *in vivo* data set, as distinct from the published *in vitro* data set. A total of 7348 Foxa1/2-bound regions (FDR <5%) that were associated with 4556 genes were discovered in the ChIP-Seq experiments, while 164 differentially expressed candidate Foxa1/2 target genes were identified by RNA-Seq experiments. As far as we know, these are the first ChIP-Seq and RNA-Seq data sets from embryonic mDA cells and therefore they provide unique and useful resources for probing molecular interactions during the differentiation of mDA cells in embryos and also from ESCs. We focused on two Foxa1/2-bound targets, namely *Neurog2* and *Smarca1*, that are unique to the *in vivo* data set. Our results have provided new mechanistic insights into how Foxa1/2 regulate the differentiation of mDA neurons.

### Foxa1/2, Otx2 and an E-box transcription factor have direct inputs into the transcriptional activity of a *Neurog2* enhancer during mDA neuronal differentiation

Previous studies have shown that the floor plate progenitors rostral to the mid-hindbrain boundary undergo neurogenesis following the activation of Neurog2 and Ascl1 in these progenitors (reviewed by Ang, 2006). Key determinants of dopamine progenitor specification, such as Lmx1a and Lmx1b (Yan et al., 2011; Deng et al., 2011), Otx2 (Omodei et al., 2008; Vernay et al., 2005), Foxa1/2 (Ferri et al., 2007) and Ferd3l (Ono et al., 2010), have been shown to be required for the expression of *Neurog2*. However, it is not known whether these genes directly regulate *Neurog2* expression. Our studies show that Otx2, Foxa2 and an E-box transcription factor directly bind to a *Neurog2* enhancer and are required for its transcriptional activity in the midbrain floor plate in mouse embryos at 12.5. These results strongly suggest that these transcription factors cooperate on cis-regulatory sequences to regulate the differentiation of mDA progenitors in the ventral midbrain. Whether the E-box transcription factor corresponds to Ferd3l remains to be determined owing to the lack of a ChIP grade Ferd3l-specific antibody.

Combinatorial requirements for Foxa, Otx2 and a floor plate-specific E-box-binding factor, such as Ferd3l, for *Neurog2* enhancer activity might explain why *Neurog2* expression in the floor plate is spatially restricted to the caudal diencephalic and midbrain regions, where all three transcription factors are expressed in mouse embryos at E12.5. However, all three factors are already expressed at E9.5, but *Neurog2* expression is only initiated at E10.75 in the rostral floor plate. This delay in transcriptional activation of *Neurog2* suggests the requirement of an additional input. Extrinsic factors are also involved in regulating mDA neurogenesis, such as Wnt (reviewed by Joksimovic and Awatramani, 2014), Shh (Lin et al., 2009; Blaess et al., 2006; Joksimovic et al., 2009; Tang et al., 2010) and LXR (Nr1h2/3) (Sacchetti et al., 2009) signalling. Analyses of the roles of these extrinsic factors will be interesting in the future, but this was not the goal of the work in this paper, which focuses on the role of transcription and epigenetic factors in mDA neurogenesis.

The change in Neurog2 expression in mDA progenitors of *NestinCre;Foxa1/2* mutants is modest compared with the effect on *Neurog2* enhancer activity when there is a mutation in its Foxa binding site. This might be due to the fact that the deletion of *Foxa1/2* occurs in *NestinCre;Foxa1/2* mutants at E11.5 and Foxa1/2 may only be required transiently for Neurog2 expression in some mDA progenitors at earlier stages. Alternatively, or in addition, other transcription factors, such as Lmx1a, Otx2 and Ferd3l, acting through different enhancers might be able to compensate for Foxa1/2 function in regulating Neurog2 expression in mDA progenitors.

### mDA neuronal differentiation involves Foxa1/2-dependent activation of *Smarca1*

Recent studies show that chromatin remodelling factors belonging to the SWI/SNF family are involved in the neuronal proliferation and differentiation of neural stem cells as well as in the developing cortex (reviewed by Hargreaves and Crabtree, 2011; Ninkovic et al., 2013). However, the role of these factors in the midbrain of developing mouse embryos has not been reported. We therefore focused on determining the role of *Smarca1* in the development of mDA neurons. We first identified *Smarca1* as a direct target of Foxa1 in mDA cells. *Smarca1* was expressed in both mDA progenitors and neurons. Despite stronger expression of *Smarca1* in progenitors than in neurons, immature neurons were generated normally, but there was a delay in the differentiation from immature to mature neurons at E12.5. This defect was rescued by E16.5. Altogether, these results suggest that *Smarca1* promotes the differentiation from immature to mature mDA neurons in the developing mouse midbrain.

The transient role of *Smarca1* might be due to compensation by other SWI/SNF factors such as *Smarca5*, which is also expressed in both mDA progenitors and neurons at this stage (data not shown). Experiments are currently on-going to determine whether *Smarca1* and *Smarca5* share redundant roles in the differentiation of mDA neurons. Independent support for the role of Smarca1 in neuronal differentiation was obtained using gain-of-function experiments in mouse epiblast stem cells. The effect of Smarca1 on neuronal differentiation *in vitro* could be due to the induction of neuronal differentiation and/or the induction of a dopaminergic (DA) phenotype in mDA neurons.

Foxa2 has previously been suggested to act as a pioneer factor in liver development and in ESC differentiation by repositioning nucleosomes (reviewed by Zaret and Carroll, 2011; Li et al., 2012). Nucleosome depletion during ESC differentiation is dependent on Nap1l1-coupled SWI/SNF and INO80 chromatin remodelling complexes. Recent studies suggest that Foxa2 and Nurr1 synergistically bind DA phenotype genes, such as *Th* and *Dat* (*Slc6a3*), during the differentiation of mDA neurons (Yi et al., 2014). In addition, Foxa2 binding to Nurr1 was shown to diminish the formation of a Nurr1-CoREST-Hdac1 repressor complex on DA phenotype genes resulting in increased histone H3 acetylation, thereby facilitating an open chromatin configuration and transcription at DA-phenotype gene promoters (Yi et al., 2014). Whether Foxa2 or Nurr1 is also involved in the recruitment of Smarca1 to DA-phenotype promoters, as has been shown for other important lineage regulators, remains to be determined in future experiments (Yip et al., 2012; Yu et al., 2013). Another possibility is that Smarca1 and other SWI/SNF factors might facilitate Foxa1/2 binding to different target sites in neurons at distinct phases of their maturation to allow for gene expression through promoting an open chromatin state at these targets. Future studies will be needed to distinguish between these different mechanisms.

## MATERIALS AND METHODS

### Establishment of Smarca1 overexpression ESC lines

ESC lines were established according to Gennet et al. (2011). Briefly, the TRE-Smarca1 expression vector was electroporated into ESCs that harbour a knock-in reverse tetracycline-controlled transactivator (rtTA) in the *Rosa26* locus (Gennet et al., 2011).

### Differentiation of ESCs

E14.1 rtTA and *Smarca1*-overexpressing ESC clones, named 2B1 and 2A4, were cultured and differentiated according to Jaeger et al. (2011).

### ChIP-qPCR

Foxa1 ChIP experiments were performed as described (Metzakopian et al., 2012). Briefly, ChIP was performed on chromatin prepared from dissected E12.5 ventral midbrain tissue using Foxa1-specific antiserum. Primers of genomic regions are listed in supplementary material Table S6. The *Foxa2* open reading frame or rabbit IgG antibody (Millipore, 12-370) was used as a negative control for non-specific enrichment. The fold enrichment of each target site was calculated as 2 to the power of the cycle. The enrichment of target sequences in ChIP material was calculated relative to input.

### ChIP-Seq and data analysis

The ChIP-Seq experiments and analysis were performed using Foxa1 and Foxa2 antibodies (Besnard et al., 2004) according to Metzakopian et al. (2012), except that mouse ventral midbrain tissue dissected from 125 wild-type mouse embryos at E12.5 was used for each experiment. Two biological replicates of each antibody were carried out. Genomic coordinates defined by MACS (Zhang et al., 2008) from the replica overlaps are provided in supplementary material Table S1. Foxa1-associated and Foxa2-associated genes were assigned by mapping peaks to the closest transcription start site of Ensembl genes. ChIP-Seq data are available at Array Express under accession number E-MTAB-3332.

### Laser capture microdissection (LCM) and RNA-Seq

E12.5 wild-type and mutant embryos were snap-frozen in dry ice-cooled isopentane. Then 10 µm cryostat coronal sections of the midbrain were cut and mounted on membrane glass slides (Zeiss 415101-4401-000) at −20°C. LCM of the floor plate was performed immediately after section mounting on slides. RNA was extracted using the Pico RNA Isolation Kit (Arcturus Engineering). The RNA-Seq library was prepared using the Ovation RNA-Seq system (Nugen). RNA-Seq libraries were sequenced on an Illumina GAIIx and analysis was performed using CLC Genomics Workbench version 1 (CLC BIO). RNA-seq data are available at Array Express under accession number E-MTAB 3333.

### Statistical analysis and functional GO analysis

Significance of enrichment between any two sets of genes was determined by applying the hypergeometric distribution as described by Gennet et al. (2011), where *x* is the number of genes shared by two gene lists (91), *n* is the number of genes identified by ChIP-Seq shared by Foxa1 and Foxa2 (1824), *M* is the number of genes of the second list (444), and *N* is the number of all Ensembl genes (37,361) (see supplementary material Fig. S1). Note that the number of genes was used rather than the probe number (entities) as provided by the microarray from Gennet et al. (2011) since multiple probes annotate some genes in the experiment. An equivalently sized list of random genes was used as a negative control. GO analysis was performed using GOToolBox (http://genome.crg.es/GOToolBox), using the MGI identities of the list of genes. Gene lists and GO term results are provided in supplementary material Table S4.

### Generation and analysis of transgenic mice

Transgenes for injection were separated from vector sequences following restriction digestion in 1% agarose gels, purified on Qiagen PCR extraction columns, precipitated and resuspended in injection buffer (10 mM Tris-HCl pH 7.5, 0.1 mM EDTA). Transgenic mice were generated by standard procedures (Scardigli et al., 2001) using fertilised eggs from CBA/Ca×C57BL/10 crosses. Transgenic embryos were identified by PCR with the *lacZ* primers (5′-3′): forward, CGAGTGTGATCATCTGGTCG; and reverse, TTACCTTGTGGAGCGACATC. E12-12.5 embryos were harvested in cold PBS and fixed for 30 min at room temperature in 4% formaldehyde in PBS (pH 7.2). Whole-mount β-galactosidase staining and analysis of the embryos were performed as described (Scardigli et al., 2001). Reporter gene expression within the developing neural tube was examined using whole-mount or 7 µm paraffin sections.

### Immunohistochemistry of brain sections

Immunohistochemistry was performed as described (Ferri et al., 2007). Quantitative immunocytochemical data represent the average±s.e.m. for cell counts of half the ventral midbrain in six consecutive sections through the entire midbrain, every 60 µm. A two-tailed Student's *t*-test was used to calculate statistical significance. The antibodies and dilutions used are described by Ferri et al. (2007) and Yan et al. (2011).

### Generation of reporter constructs

Regulatory sequences beneath Foxa1 peak regions called by MACS were selected according to the limits defined by the highest sequence conservation among multiple vertebrate species from the UCSC genome browser (http://genome.ucsc.edu/). All regulatory sequences assayed were cloned into the *Xba*I site of a reporter vector comprising the β-globin promoter, *lacZ* cDNA, SV40 large T-antigen poly(A) site and sequences mediating *Neurog2* enhancer activity in an arrangement that retains the native orientation. The DNA sequence tested was in chromosome position chr3:127,349,185- 127,349,631 and was generated by PCR amplification using primers (5′-3′): forward, TCTAGAAATCTGTAAACCCATCACATG; reverse, TCTAGAAGAGGCCACCCTCTCCCCTC. Point mutations designed to disrupt DNA binding at the recognition sequences for Otx2, Foxa1 and the E-box were performed using the QuikChange Mutagenesis Kit (Agilent Technologies) with the following primers (5′-3′): Otx2, GGGCGGGGGCTTAGAAAAAGTCTCGGCTCTTCATGAATAG; Foxa1, GATGAGTGCCGACGGCGTACCCACCTTCATGAGCTAAAAG; E-box, CGTCATCTCAAAGCCGCATCTTTGCCATTGAGCCGAAAAG (mutated residues are underlined). The integrity and orientation of all constructs containing PCR-generated fragments was confirmed by DNA sequencing.

### Generation and genotyping of mutant embryos and animals

All mouse strains were maintained in a mixed MF1-129/SV background. *NestinCre/+*, *Foxa2^flox/flox^* and *Foxa1^loxp/loxp^* mouse strains were generated as described (Ferri et al., 2007; Gao et al., 2008). Here, we refer to the *Foxa1^loxp^* allele as *Foxa1^flox^*. *Foxa1^flox/flox^;Foxa2^flox/flox^* mice were generated by crossing *Foxa1^flox/flox^* with *Foxa2^flox/flox^* animals. To obtain conditional *Foxa1* and *Foxa2* double mutants, we first crossed *NestinCre/+* mice with *Foxa1^flox/flox^;Foxa2^flox/flox^* animals. Subsequently, *NestinCre/+;Foxa1^flox/+^;Foxa2^flox/+^* F1 males were mated to *Foxa1^flox/flox^;Foxa2^flox/flox^* females to generate *NestinCre/+;Foxa1^flox/flox^;Foxa2^flox/flox^* double mutants. The *Foxa2^flox^* and *Foxa1^flox^* alleles (Gao et al., 2008; Hallonet et al., 2002) and *Cre* transgene (Indra et al., 1999) were detected by PCR as described. *Foxa1^flox/flox^;Foxa2^flox/flox^* embryos were used as control embryos in all experiments. Animals were handled according to the Society of Neuroscience Policy on the Use of Animals in Neuroscience Research, as well as the European Communities Council Directive.

### RNA extraction, cDNA synthesis and RT-qPCR analyses

Ventral midbrain tissue was dissected from three *Foxa1^flox/flox^;Foxa2^flox/flox^* control embryos and three *NestinCre;Foxa1/2* cko embryos. cDNA synthesis and RT-qPCR analyses were performed according to Metzakopian et al. (2012). Primers are provided in supplementary material Table S6.

### Immunohistochemistry of differentiated ESCs

Cells were fixed in 4% PFA for 10 min at room temperature. Primary antibody was added and cells were incubated at 4°C overnight. Cells were then washed three times with PBS containing 0.1% Triton X-100. Secondary antibody was added and cells were incubated at room temperature for 1 h. Antibodies used: sheep anti-Th (Millipore, AB1542; 1:1000) and mouse anti-Tuj1 (Tubb3; Millipore, MAB1637; 1:1000). For quantitative analysis, DAPI-stained colonies were chosen randomly from three independent experiments of each condition and the percentage of cells expressing Th was determined. Two-tailed Student's *t*-test was used to calculate statistical significance.

### *In situ* hybridisation of brain sections

Section *in situ* hybridisation of E12.5 mouse brains was performed as described (Lin et al., 2009). The following probes were used: *Smarca1*, *Dmrtb1* and a PCR fragment corresponding to the first 600 bp of *Lmcd1* (NCBI gene ID: 93761, 56296 and 30937, respectively).

## Supplementary Material

Supplementary Material
