# Supplementary material for: Genome-wide characterisation of Foxa1 binding sites reveals several mechanisms for regulating neuronal differentiation in midbrain dopamine cells
Source: Development. 2015 Apr 1;142(7):1315–24. doi: 10.1242/dev.115808 (PMC4378246; doi:10.1242/dev.115808)
Supplement: Supplementary Material [file supp_142_7_1315__index.html]

Supplementary Material 

# Genome-wide characterisation of Foxa1 binding sites reveals several mechanisms for regulating neuronal differentiation in midbrain dopamine cells

## DEV115808 Supplementary Material

**Files in this Data Supplement:**

- Supplementary Material
