# Supplementary material for: Genome-wide characterisation of Foxa1 binding sites reveals several mechanisms for regulating neuronal differentiation in midbrain dopamine cells
Source: Development. 2015 Apr 1;142(7):1315–24. doi: 10.1242/dev.115808 (PMC4378246; doi:10.1242/dev.115808)
Supplement: Supplementary Material [file supp_142.7.1315_DEV115808supp.pdf]

A

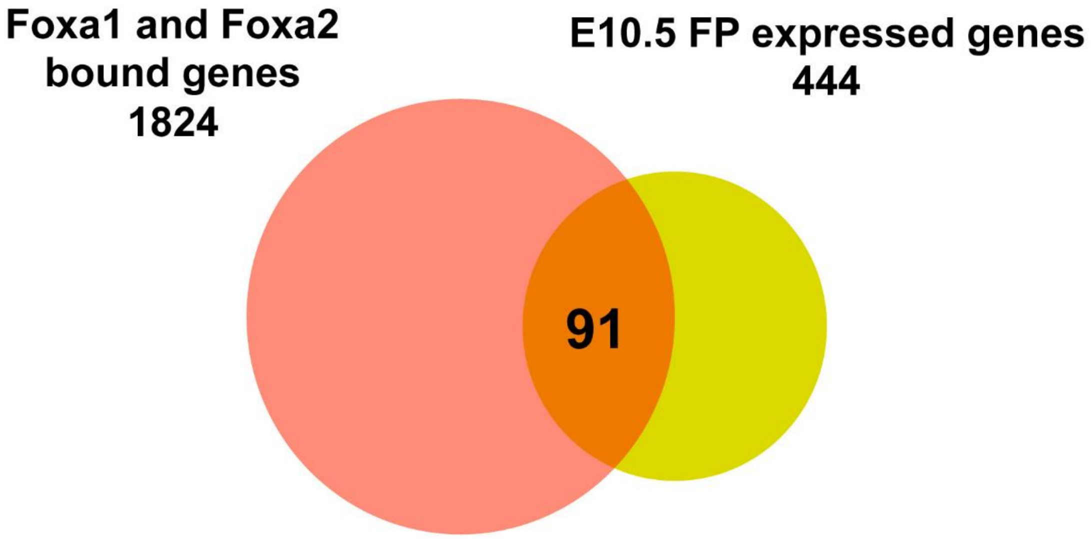

B

Data set	FP	Random
Shared	91	29
p-value	6.86E-31	0.015

Fig. S1. Venn diagram showing the overlap of Foxa1/2-bound genes and those preferentially expressed in the midbrain floor plate at E10.5 (A) and statistical analysis of the shared genes in A and the same number of randomly selected genes (B).

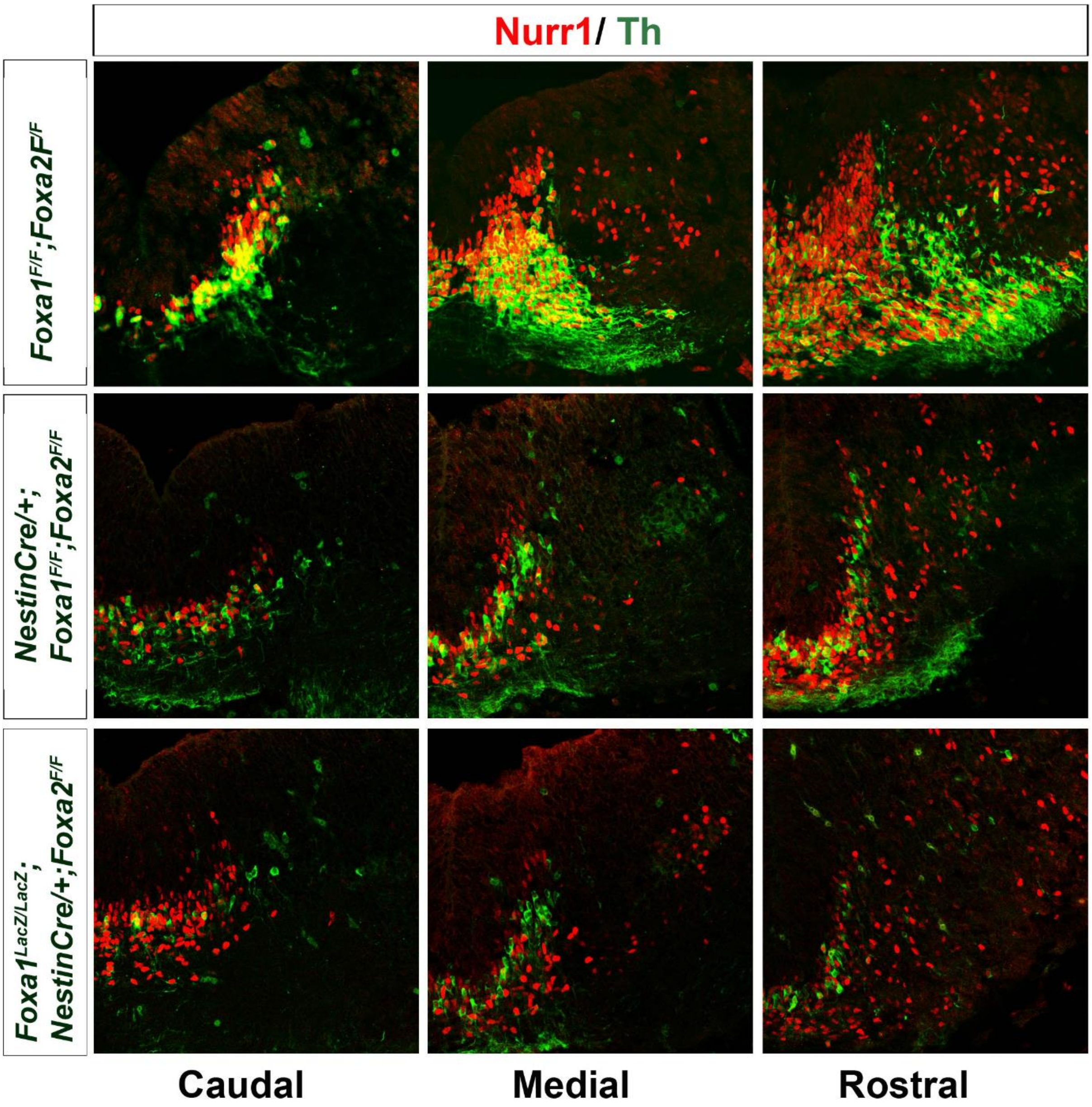

Fig. S2. A similar reduction was observed in the number of Nurr1+ and Nurr1+, Th+ mDA neurons in coronal sections through rostral, medial and caudal levels of the ventral midbrain of *NestinCre;Foxa1*$^{F/F}$*;Foxa2*$^{F/F}$ and *Foxa1*$^{lacZ/lacZ}$*; NestinCre;Foxa2*$^{F/F}$ compared with control *Foxa1*$^{F/F}$*;Foxa2*$^{F/F}$ embryos at E12.5.

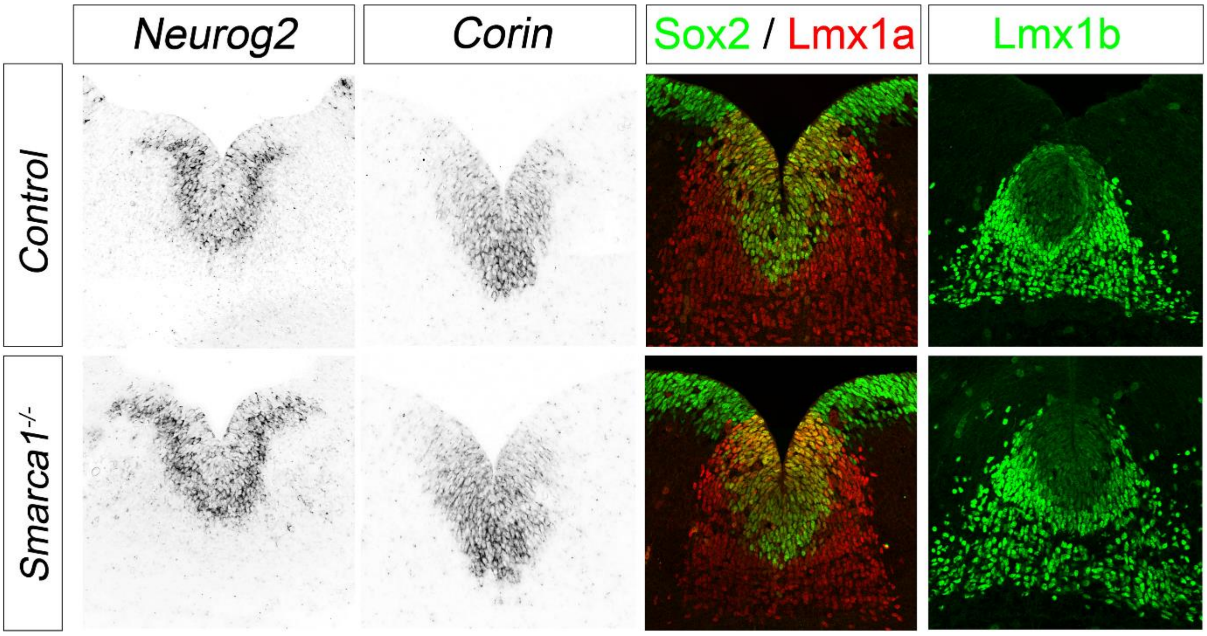

Fig. S3. Expression of mDA progenitor markers by *in situ* hybridisation and by immunohistochemistry was similar in coronal sections of the midbrain of control and *NestinCre;Foxa1/2* cko embryos at E12.5.

Table S1. Foxa1 ChIP-Seq peaks called by MACS. Data include: genomic coordinates, tag number, fold change, *P*-value, false discovery rate and closest TSS.

Click here to Download Table S1

Table S2. Foxa1 *in vivo* and Foxa2 *in vitro* co-bound genomic regions annotated to the closest TSS of genes expressed in mouse midbrain floor plate.

Click here to Download Table S2

Table S3. Overlap of differentially expressed genes identified by *in vivo* RNA-Seq analysis of wild-type versus *Nestin:Foxa1/2* cko ventral midbrains with genes associated to *in vivo* Foxa1 ChIP-Seq peaks. MGI identification codes and enriched Gene Ontology terms are also provided here.

Click here to Download Table S3

Table S4. Genomic coordinates discussed in the main manuscript including PCR primers used in ChIP-qPCR validation. Primers used in expression qPCR analysis are also mentioned here.

Click here to Download Table S4

Table S5. Foxa2 ChIP-Seq peaks called by MACS and their overlap with *in vitro* Foxa2-bound and *in vivo* Foxa1-bound genomic regions.

Click here to Download Table S5

Table S6. Overlap of Foxa2 *in vivo* ChIP-Seq peaks with Foxa1 *in vivo* ChIP-Seq peaks.

Click here to Download Table S6
